# David Curtis

**DOI:** 10.1192/bjb.2023.72

**Published:** 2023-12

**Authors:** Abdi Sanati

**Figure:**
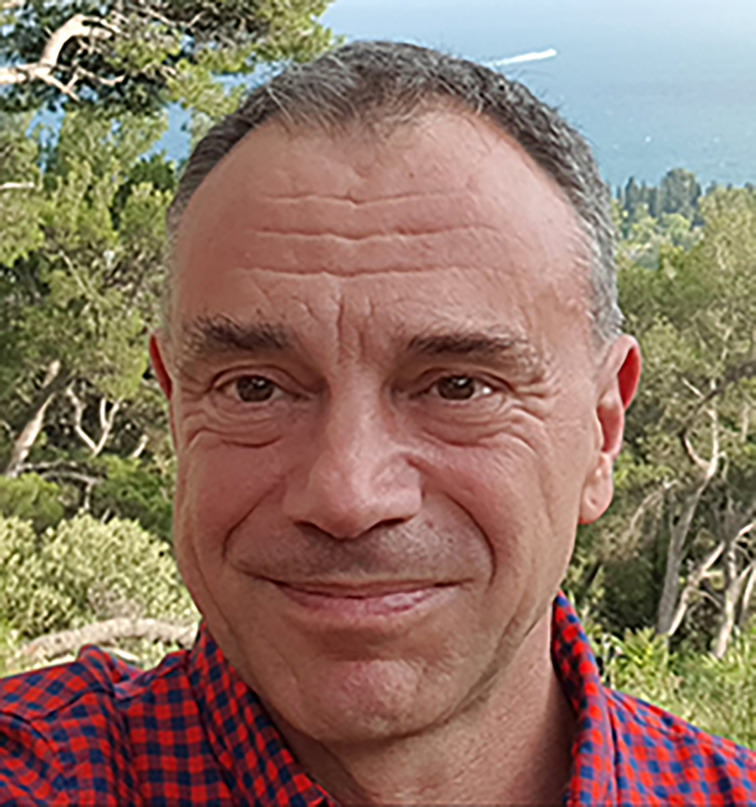

Professor David Curtis is an honorary professor at UCL Genetics Institute and a retired consultant psychiatrist. He has had hundreds of publications in peer-reviewed journals and has been one of the pillars of psychiatric genetics. I first met him when attending the educational meetings at St Bartholomew's Hospital, London. I admired his uncompromising stance when it came to scientific rigour. You did not want to present a substandard paper before him! Over the years I got to know him more and found it a learning experience. He is someone you could disagree with but never ignore.

**After years of practice, I have come to the view that when non-geneticists start giving an opinion on genetics, I become very sceptical. And I was thinking, why do you think that knowledge of genetics among general psychiatrists is not too good?**

Well, I think one reason is that until recently, there hasn't been any need for psychiatrists to know very much about genetics. Outside of dementia or intellectual disability, there hasn't been much useful knowledge. It's been perfectly legitimate for psychiatrists to be fairly ignorant about genetics, because there hasn't been anything important that anybody needed to know. That has changed now. But I wouldn't criticise. It's not only psychiatrists, it's doctors in general who haven't needed to know much about genetics. But there have been huge changes in genetic technology over the last few years, which means that now it does come more to impact on medicine and psychiatry. I think it's a big challenge for the whole of medicine, that people who trained years ago who are experts in their profession face this whole lot of new, difficult science. There are also a lot of conflicting voices. It's very difficult for outsiders to know what's real, what's not real, what's important, what's not important. There's a lot of partisan stuff coming from different places. I think it's very difficult for people to have a good understanding of the things they actually need to understand.

**I remember, a few years ago, at one of the RCPsych conferences, one very prominent scholar was using genetics to prove that psychosis was in continuity with normality. You rightly corrected me that you cannot come to this conclusion from genetics. My worry was that sometimes genetics is used to push hypotheses that cannot be based on genetics.**

I think there are all sorts of problems and caveats. People can use genetics to promote whatever they want to promote. I think, in psychiatry and genetics in general, people are very concerned about the possibilities for different kinds of abuse, for genetic findings to be used to prop up hypotheses which are not actually true and for genetic practices to be misused. What you state is one kind of example of that. Genetics doesn't prove that schizophrenia is on a spectrum with normality. In fact, I would argue that what we're seeing from our genetics research is kind of quite the opposite. It's very much in accordance with the medical model of an illness. There are genetic risk factors for an illness and then people can develop an illness, in the same way as we see in all sorts of places in medicine.

**I talk to people who, like me, are not well versed in genetics and their approach is problematic. For example, they state that because you can't find a particular gene for a problem, that means it doesn't exist, which is treating genes like biomarkers.**

It's a very complex situation. What is the significance of saying that there is some genetic contribution to a condition? What are the implications of that? How is the implication of that different from saying that we know that if this gene is abnormal, then it dramatically increases the risk of a condition? Those two kinds of statement have two very different sets of implications. I think most people are really not all that uncomfortable with an idea that there's a genetic contribution to things. We take it for granted that people have intrinsic differences. Sigmund Freud was perfectly happy with the idea that not everything was environment and upbringing, and people had individual innate temperaments. I think by and large, not many people are complete blank slaters, believing nothing is innate to somebody. But then the interesting stuff from the medical point of view is from the single genes, where you can say that if this gene is abnormal it has a big effect on that disease. And then we can start asking, well, why is that? What's that telling us about the nature of that disease? What does that gene do? What does that protein do? How might that give us a handle on what's going wrong? How might that help us to treat that disease better? And that's really the bit of genetics I'm interested in. That kind of knowledge has proved very, very helpful.

**Do you think genetics has been overlooked in favour of other factors, such as psychosocial or biological non-genetic factors?**

I wouldn't say overlooked. I think people acknowledge contributions from all sorts of different kinds of risk. The genetic kinds of risk or environmental exposures are more important in some conditions than others. I think that's all fair. Within medicine there's not a lot of people that would say there's no role for genetics in anything. I think maybe outside of medicine, within academic sociology or academic psychology, not clinical psychology, some people might say that. I think the general consensus of medical and psychiatric thinking is that there are different kinds of risk factor that can all contribute to conditions and it's not one or the other. They're not in competition, they seem to come from different places.

**One of the new paradigms that is very dominant is trauma. I have been fearful that sometimes other factors are overlooked, because I've seen people sense that everything could be explained by that.**

I think people who say that are wrong. It's a very unconvincing notion intuitively for psychiatrists, that every mental disorder is some response to adverse environment. We've all seen many patients who've had very severe mental illnesses and had perfectly normal childhood backgrounds. We can also, obviously from research, see that trauma doesn't explain everything. There will be people that exaggerate the role of trauma. I don't think that's helpful.

**Do you think genetics could help us in the future to identify people who could respond to particular treatments and guide us?**

This is a very interesting, very topical question, which applies in psychiatry, but also applies in the rest of medicine. There's this idea about an application for genetics, which is precision medicine – that you'll be able to do a blood test on somebody and, based on their genetics, give them the right treatment for their blood pressure or their schizophrenia. My position on that, not just in psychiatry but for medicine in general, is that it is not going to work. Basically, for all these conditions – diabetes, high blood pressure, high cholesterol, schizophrenia – what we see is that there are a very small number of people that have an identifiable genetic abnormality which gives them their illness. Essentially that has a really strong effect in those people. Now, there are two or three things about that. One is that 95% of patients with any particular diagnosis will not have a clear genetic cause. So for most people, it won't work at all. Even for the people whose illness does have a genetic cause, when you can say they've got diabetes because they've got that abnormality of that gene, often that doesn't mean that you give them a different treatment. In familial hypercholesterolaemia, somebody has a genetic abnormality that gives them very high lipids and you give them the same treatment. You don't necessarily give them a different treatment because of which gene is involved. So even when you can identify the genetic abnormality, it may not help. The third thing is about how we get that knowledge. So if you say doing a genetic test would help you to give this person a better treatment, how are you going to prove that? Where's the evidence going to come from? To get evidence, you have to do a trial where you give some people a genetic test and others no test and use the treatment. You have to show that the people who had the test get better results. You have to show that people with this genetic profile do better on this treatment, and people with that genetic profile do better on that treatment. How are you going to design a study to do that? If you think about doing a drug trial, it is expensive just to prove the drug works at all. It costs a billion dollars to get a drug approved. So to prove that a genetic test works, and helps you to treat, you're talking about huge investment and hundreds or maybe thousands of patients in a trial. That is never going to happen. A few weeks ago a trial was published in *The Lancet* on doing genetic testing to ask ‘Can we use genetic testing to help decide what dose of treatment somebody should have?’ It was a big trial with thousands of people across multiple centres. The people who did the trial claimed that it showed how great the testing was. Then a few weeks later, *The Lancet* published three or four letters, and one of them was from me, which said actually the trial doesn't show that at all. So the biggest trial there has ever been in medicine on genetic testing shows that it actually doesn't help. So I do not think that genetic testing is going to have any kind of role in medicine in general, except for a few very specific cases. Genetic testing in cancer is important. Using genetics to guide cancer treatment is helpful. There'll be one or two other examples. But by and large, genetic testing is not going to be something that doctors will be using to give better treatment to their patients.

**If I may go a bit philosophical I want to ask you this. I was reading that some people say that genetics leads to some form of determinism and denial of freedom of will. What do you think?**

I think there are two sides to this coin. If you say to a patient ‘You've got genes which say you're at a high risk of having a heart attack’ does that mean that the patient says ‘Oh, gosh, I'm at high risk, I'd better be careful about my diet, exercise and not smoking’? Or does the patient say ‘The genes will give me a heart attack and it doesn't matter what I do, I may as well smoke because I'm going to get the heart attack anyway’? If you tell somebody that they're at low risk of an illness, does that mean that they say ‘Oh, then it doesn't matter, I'll smoke and drink as much as I want because I'm at low risk of illness’. Or not? One of the arguments for trying to do genetic research in psychiatry is to remove stigma. The idea is we demonstrate that schizophrenia is a real illness like epilepsy, like diabetes. And then people can say ‘It's because I've got this illness, it's not my fault. I've got a real medical illness. Maybe I have some genetic abnormalities that make me prone to this. It's not my weak willpower. My friends are telling me I should be able to manage without medication, but now I know I've got a real illness. And so I should be taking medication’. So that's a view where the idea of the genetics being the basis of illness is to try to reduce stigma. But then the other way around is, people could say they have a genetic propensity to the illness and it is something they are stuck with and cannot do anything about. It has implications for the family and children, and that may increase stigma. It is a legitimate controversy in psychiatry, in genetics and in medicine. Is identifying a genetic contribution to something empowering, as we would hope it would be? Or as you suggested, may people go down the route of genetic determinism, saying they are at mercy of their illness and can do nothing about it? It would rob them of their autonomy, freedom to make choices and so on. It's a controversy and people have done some research into this. And by and large, it's not too strongly one way or the other, but people recognise that there can be risks in how genetics is perceived. I suppose a major part of the risk is that people think genetics is more important than it really is. Most genetic results are really not very important at all. So that tends to be the direction of the mistake people make. There is an increasing awareness in the genetics research community that we have to be very careful about how we talk about results. Just because something's a ‘bit genetic’ doesn't mean that you can't do anything about it. Real genetic determinism is incredibly rare. There are very few things that actually are strongly determined by genes.

**What do you say to people in the conversations about genetic testing who bring up eugenics?**

This is one of the major concerns. Where would this be going? How will people be using this? How will people view it? When I talked earlier about the possibility for these things to be misinterpreted and misused, very much on our minds is the idea of eugenics and the potential for abuse. And indeed, we've published fairly recently an article expressing our concerns about the way tests might be misused for testing fetuses. And there are also concerns about misuse of tests for babies. Tests are commercially sold that are supposed to give you a better child. There are controversies about the extent to which people should or should not be doing prenatal testing to try to select better embryos. I think it's fair to say that most people would be against certain kinds of testing. And we've argued against it. But there are voices, what we would call eugenicists, out there saying that this kind of test would be helpful. It's a very complex, difficult and alarming issue. I think people outside the profession can legitimately claim that there's cause for concern. So we have to be very careful about how we talk about this kind of research, this kind of knowledge and what we do with it. I was at the World Congress of Psychiatric Genetics recently, and there were groups of protesters outside who have the view that the idea of psychiatric genetics is getting close to eugenics and there are all sorts of possibilities for misuse. In terms of being able to keep people on side, I know that there was recently a research programme which was supposed to be launching research into autism. People with autism were very concerned about the direction the research was taking and protested it. So I think it would absolutely be a mistake to dismiss concerns about eugenics and say this has nothing to do with eugenics. I think instead what we have to do is to say yes, we have to be very clear about why we are doing this. What are the benefits? What will we be doing with it? What is valid? What is not valid? We need to think about what we do very carefully and be cautious and sensitive about those concerns, because I don't think those concerns are completely unrealistic. It's not fantasy, we have to be concerned about how genetic knowledge can be used and misused, and also how it can be misrepresented and then misused.

**Just one last question. Recently going through the papers I saw that you withdrew your subscription and contributions to *The Lancet* because they referred to women as ‘bodies with vaginas’. Do you think that medicine has been too cautious in this debate?**

I think a lot of stuff has come up. I think it's a bit like genetics, there's a lot of stuff going on that a lot of doctors are not really very aware of. There have been some bad actors that have promoted particular views, which are not evidence based and not thought through. And people have instinctively tried to be kind, inclusive and sensitive, because they're nice people, and have actually got into ridiculous, unscientific, indefensible, illogical positions, which help absolutely nobody. And I think it's very important that the doctors should stand up for biological reality. Let's take a real concrete example. In psychiatry, supposing you're a psychiatrist and you're seeing a patient, an adult patient who's 40 years old. You're going to take a psychiatric history. It would to me be absurd to not know that that person had gone through, say, gender reassignment surgery when they were 19. If you're trying to understand somebody, to not know that they spent 19 years as a man and then the next 20 years a woman, to me that's absurd. The idea that this piece of information about somebody's birth sex is so sensitive that the medical team looking after them should not be aware of it – to me that's clearly nonsense. And yet that seems to be the direction that some people would be heading in. The idea that in somebody's medical records, you cannot include what their biological sex is, it's nonsense. The idea that to avoid hurting some people's feelings, we don't say women, and because it somehow might be seen as being exclusionary, we say ‘bodies with vaginas’, it's offensive, sexist, paternalistic and regressive. It is not helpful to anybody. It provokes a kind of a backlash, which is unhelpful. And, you know, for someone to use such language one has to be absolutely tone deaf. One has to be, I think, kind of pretty sexist and pretty unthinking. So, yes, to me, that's just offensive language. The apology that Richard Horton gave was kind of mealy mouthed. I thought this is not an institution that I would be associated with. It doesn't represent the values that I would want to uphold.

